# Oscillating expression of interleukin-16 in multiple myeloma is associated with proliferation, clonogenic growth, and PI3K/NFKB/MAPK activation

**DOI:** 10.18632/oncotarget.17534

**Published:** 2017-04-28

**Authors:** Julia Templin, Djordje Atanackovic, Daniel Hasche, Sabarinath Venniyil Radhakrishnan, Tim Luetkens

**Affiliations:** ^1^ Division of Hematology and Hematologic Malignancies, Huntsman Cancer Institute, University of Utah, Salt Lake City, UT, USA

**Keywords:** multiple myeloma, cytokines, tumor biology, tumor immunology, interleukin-16

## Abstract

Multiple myeloma (MM) is an incurable hematologic malignancy emerging from a plasma cell clone located in the bone marrow and is characterized by a high rate of fatal relapses after initially effective treatment. We have previously identified Interleukin-16 (IL-16) as an important factor promoting the proliferation of MM cells. We demonstrate here an upregulated, periodic expression, and secretion of IL-16 by MM cells leading to high extracellular IL-16 levels. The level of IL-16 released from a given MM cell line correlated with its proliferative activity. Establishing an inducible knockdown system and performing gene expression arrays we observed an association between IL-16 expression and activation of PI3, NFκB and MAP kinase pathways and, specifically, genes involved in tumor cell proliferation. Functional assays showed that IL-16 knockdown reduced the proliferative activity with a significant delay in cell cycle progression to G2 phase of conventional MM cells and completely suppressed the growth of clonogenic MM cells, which are suspected to be responsible for the high relapse rates in MM. Overall, our results demonstrate that tumor-regenerating MM cells may be particularly susceptible to IL-16 neutralization, suggesting an important role of anti-IL-16 therapies in the treatment of MM, particularly in combination with existing strategies targeting the bulk of myeloma cells.

## INTRODUCTION

Multiple Myeloma (MM) is a plasma cell malignancy arising in the bone marrow (BM) which causes failure of myelopoiesis, renal insufficiency, osteolytic lesions, and immune dysfunction. Therapeutic options for MM have improved over the past decade, however, cures still represent a rare exception and most patients will succumb to the malignancy within 6 years after diagnosis [1]. Thus, there is an urgent need to identify and target molecular factors contributing to tumor progression and the occurrence of therapy resistance.

Myeloma strongly depends on its local environment in the BM. The tumor microenvironment of MM comprises hematopoietic stem cells, stromal cells, endothelial cells, osteoclasts/osteoblasts, and a variety of immune cells. Interactions of MM cells with accessory cells in the tumor milieu trigger tumor cell survival and growth, angiogenesis, immunosuppression, and drug resistance [2–8]. Key players identified are growth factors IL-6, IGF-1, and VEGF [9, 10], as well as surface molecules CD40-CD40L, VCAM-VLA4, and MUC-ICAM [11–13]. These molecules mediate their tumor-promoting effects by stimulating the PI3 kinase, NFκB, and MAP kinase pathways [9], which are signal cascades central to the development and progression of MM [14].

Very recently, we have described for the first time cytokine Interleukin-16 (IL-16) as a myeloma-promoting factor [15]. In the physiological context, IL-16 is produced as a precursor molecule and secreted by T cells [16] and other leukocyte subsets [17, 18] after processing and cell activation. It acts as a factor chemoattracting various types of immune cells and regulating their activation status [19]. Within the past few years, IL-16 has been reported to be overexpressed in different solid and hematologic tumors [20–24]. In myeloma, plasma levels of IL-16 seem to correlate with the severity of the disease and the patients' prognosis [10, 25–27]. IL-16 concentrations in the BM of myeloma patients correlate with the number of malignant plasma cells and we have shown for the first time that the tumor cells themselves produce this cytokine. In addition, we obtained preliminary data supporting a role of IL-16 in promoting the proliferation of myeloma cells [15].

In the present study, establishing a stable and inducible knockdown system and thereby minimizing the effect of varying mRNA expression on knockdown efficiency and functional assays, we confirmed the important role of IL-16 as a proliferation-inducing cytokine in MM and we further substantiated this finding by performing whole genome expression analyses. Most importantly, we show here that myeloma-propagating cells become entirely unable to form colonies in the absence of IL-16 suggesting that tumor-regenerating myeloma cells may be particularly susceptible to IL-16 neutralization. Overall, our results indicate an important role of anti-IL-16 therapies in the treatment of MM, in particular in combination with existing strategies targeting the bulk of myeloma cells.

## RESULTS

### Multiple myeloma cells constitutively express IL-16 in a periodical pattern and secrete soluble IL-16 protein

**Figure 1:**
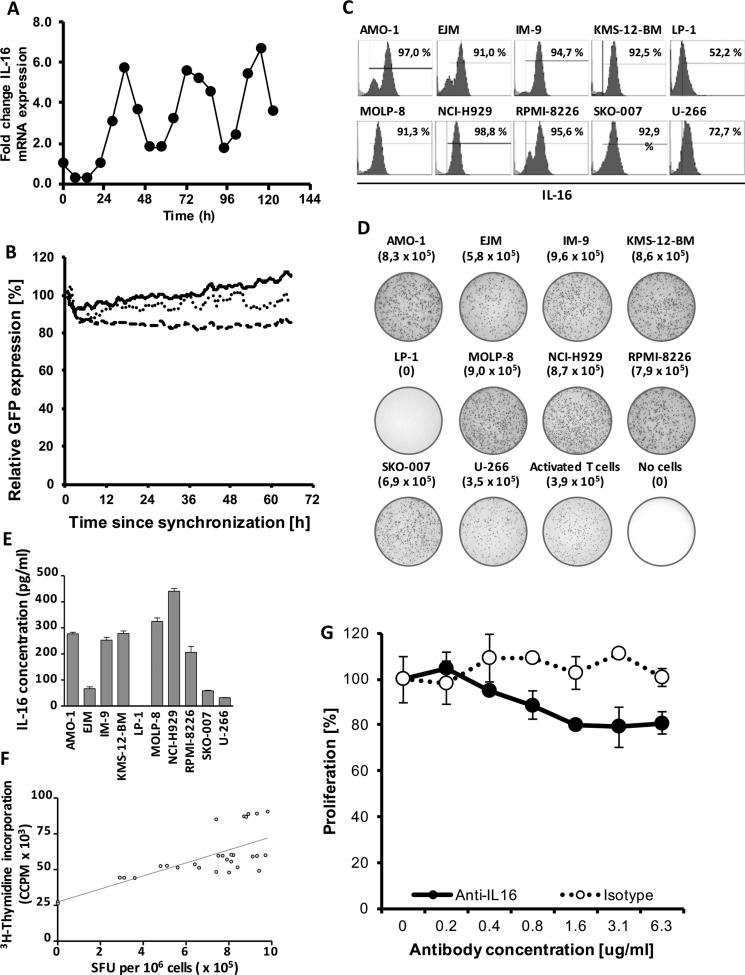
Stable downregulation of oscillating IL-16 mRNA expression (**A**) Representative quantitative RT-PCR of IL-16 expression in RPMI-8226 cells after normalization to GAPDH. Cells were synchronized for 24 h by serum starvation and then grown under standard conditions. (**B**) Cumulative fluorescence intensity in RPMI-8226 cells of three destabilized GFP mutants expressed under control of the human IL-16 promoter as determined by spinning-disk live cell fluorescence microscopy. The solid line represents MM cells expressing pTurboGFP-dest1, the dotted line represents cells expressing pTurboGFP-dest1 including the mutation H423A, and the dashed line represents cells expressing pTurboGFP-dest1 including the mutations D433A and D434A. Lines represent a moving average of 4 time points. (**C**) Intracellular staining of IL-16 in MM cell lines as determined by flow cytometry. (**D**) IL-16 ELISpot demonstrating strong IL-16 secretion in 9 of 10 MM cell lines as well as activated primary human T cells. (**E**) IL-16 concentrations in culture supernatants harvested from 10 MM cell lines as determined by ELISA. (**F**) Correlation of Spot-Forming Units (SFU) as determined by IL-16 ELISPOT and MM cell proliferation as determined by ^3^H-Thymidine incorporation assay. (**G**) Proliferation of MM cell line RPMI-8226 after 12 h treatment with an IL-16 neutralizing antibody or an isotype control antibody as determined by ^3^H-thymidine incorporation assay.

We have previously shown that myeloma cells strongly express IL-16, however, it has remained unclear whether only a certain fraction of the tumor bulk is producing this cytokine at any given timepoint and/or whether there are temporal variations in IL-16 synthesis. Therefore, in a first step, we assessed the mRNA expression pattern of MM cells synchronized by serum starvation to determine variations in IL-16 expression levels over time. Interestingly, we indeed observed periodical fluctuations in IL-16 mRNA expression (Figure 1A) showing an approximately circadian regulation of IL-16 expression in MM cells. We next asked the question whether these changes were related to varying promoter activity. Using confocal time-lapse imaging of RPMI-8226 cells expressing three increasingly destabilized GFP variants [28] under control of the human IL16 promoter [29]. We found, however, that promoter activity was comparably stable suggesting that IL-16 mRNA levels are regulated post-transcriptionally (Figure 1B). Exploring whether these time-dependent variations would translate into differences in IL-16 protein expression in MM cells, we next analyzed levels of intracellular IL-16 protein in 10 different MM cell lines and we found that in the case of 8 of these lines more than 90% of the cells were positive for intracellular IL-16 (Figure 1C) indicating a constitutive and homogenous protein expression of this cytokine in MM.

Next, we asked the question whether we would also find secretion of IL-16 by the majority of tumor cells at any given timepoint and we used an ELISPOT assay to determine cytokine release on a single cell level. While under physiological conditions normal leukocytes only secrete IL-16 upon cell activation [16], we found that in 9 out of 10 MM cell lines about 80% of all cells actively secreted IL-16 even without prior activation (Figure 1D). This constitutive secretion of IL-16 by most myeloma cell lines resulted in substantial levels of the cytokine in culture supernatants (Figure 1E).

### Secretion of IL-16 correlates with the proliferative activity of MM cells and IL-16 silencing results in the downregulation of genes promoting cellular proliferation

**Figure 2:**
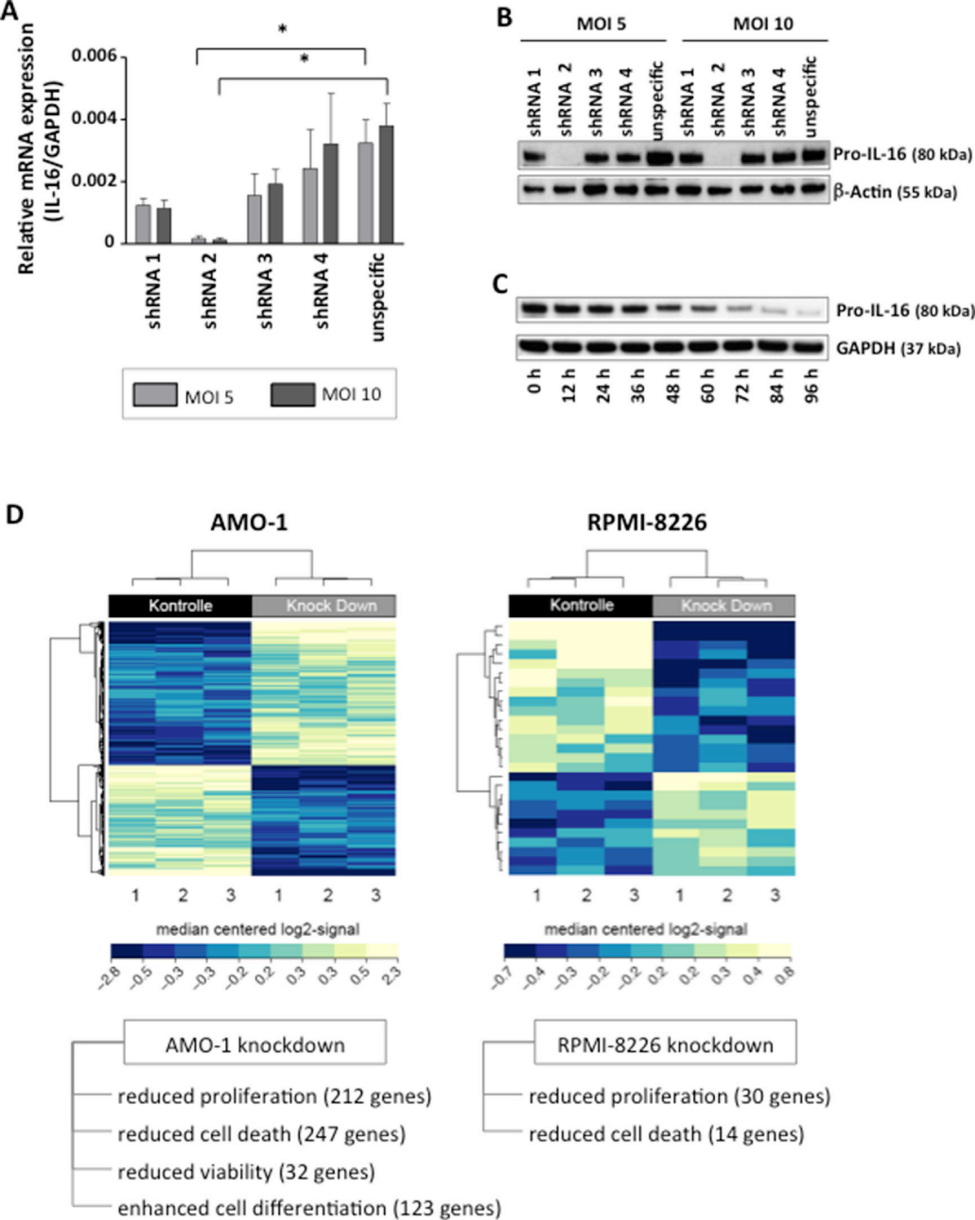
Downregulation of IL-16 in MM cell lines and genome-wide mRNA expression analysis (**A**) Relative expression of IL-16 mRNA as determined by quantitative RT-PCR in MM cell line RPMI-8226 after induction of IL-16-specific or control shRNA after transduction of cells with lentiviral supernatants at different multiplicities of infection (MOI). (**B**) Western blot of Pro-IL-16 and loading control β-Actin at 96 h after transduction with four IL-16-specific shRNA constructs or one unspecific control shRNA. (**C**) Time-course of the expression of Pro-IL-16 and loading control GAPDH over 96 h after transduction with IL-16-specific shRNA. (**D**) Heatmap of hierarchically clustered genes differentially regulated in MM cell lines expressing IL-16-specific shRNA or control shRNA.

Our transient knockdown studies had previously indicated a role of IL-16 in promoting proliferation of myeloma cells [15] and in our current study we asked the question whether there was an immediate association between the secretion of soluble IL-16 and the proliferation of the respective cells. Interestingly, we indeed observed a strong positive correlation (*R*^2^ = 0.557; *p <* 0.0001) between the number of IL-16 producing cells in a given culture, as measured by ELISPOT and the proliferative activity of the respective MM cell line (Figure 1F). We next aimed to determine whether soluble IL-16 promotes MM cell proliferation through autocrine signaling. Indeed, blocking of IL-16 using a neutralizing antibody led to a dose-dependent reduction in the proliferative activity of MM cells compared to an isotype control antibody as determined by ^3^H-thymidine incorporation (Figure 1G). In order to further assess the functional consequences of IL-16 expression and secretion in MM we next developed and optimized a system for the stable knockdown of this cytokine in myeloma cells. We previously used a siRNA-based system for the knockdown of IL-16 [15], however, in our more recent analyses a stable integration of inducible shRNAmir by lentiviral transduction turned out to be the most reliable system for IL-16 gene silencing. We found that 1 out of 4 shRNAmir sequences tested led to a 98% reduction of IL-16 mRNA and protein expression (Figure 2A and 2B) with the highest knockdown efficiency at 96 h after induction (Figure 2C).

Performing a genome-wide expression analysis we next analyzed the effect of IL-16 knockdown on differential gene expression. In both MM cell lines tested, silencing of IL16 caused a differential regulation of gene expression (Figure 2D). In particular, we observed reduced expression of genes promoting cellular proliferation and apoptosis as a consequence of IL-16 knockdown. In addition, in MM cell line AMO-1 we observed differential expression of genes involved in cell viability and differentiation.

**Figure 3:**
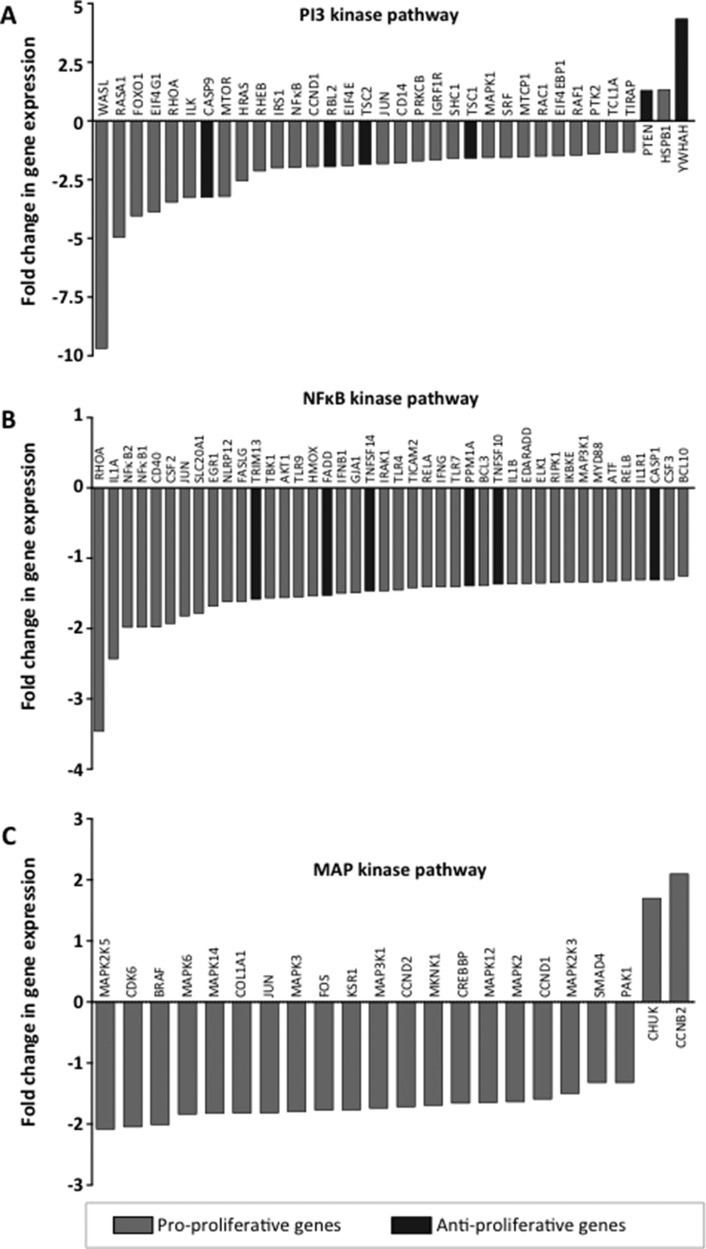
Expression of pathway-specific genes after IL-16 knockdown Quantitative RT-PCR assays were used to validate changes in mRNA expression in RPMI-8226 cells treated with IL-16-specific shRNA over cells expressing control shRNA. Genes are identified as pro-proliferative and anti-proliferative according to their respective gene ontology annotation [45]. Genes are clustered into the three major molecular pathways (**A**) PI3K, (**B**) NFκB, and (**C**) MAPK.

In order to identify molecular mechanisms utilized by IL-16 to support cellular growth we next analyzed three major pathways (PI3 kinase, NFκB, MAP kinase) known to promote the progression of MM [14, 30] after knockdown of IL-16 in RPMI-8226 cells. In agreement with our findings obtained by genome-wide expression analysis, our pathway-focused analyses showed a reduced expression of a large number of genes promoting proliferation across all three molecular pathways (Figure 3). Involved were a number of key proteins such as mTOR, Ras, NFκB1, NFκB2, Jun, Fos, and several MAP kinases. In addition, we observed within the PI3k pathway an upregulation of genes PTEN and YWHAH, which are known to inhibit proliferation (Figure 3A).

### IL-16 promotes proliferation of bulk and clonogenic MM cells

**Figure 4:**
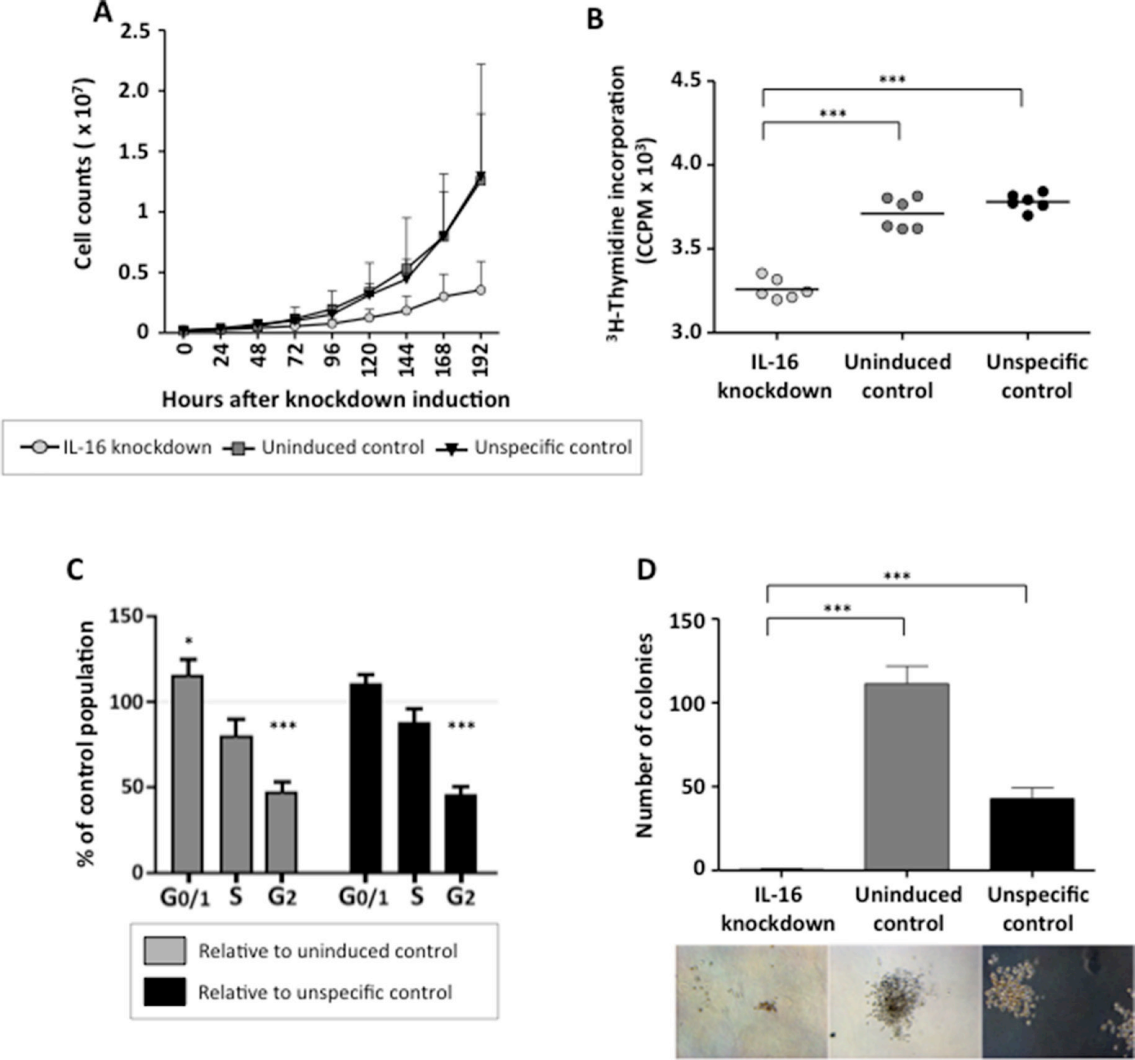
Effects of stable IL-16 knockdown on MM cell proliferation and clonogenic growth (**A**) Growth curve of MM cell line RPMI-8226 either uninduced or induced to express an IL-16 specific shRNA or a control shRNA. Values represent the mean of 3 independent experiments. Error bars represent the standard deviation of 3 independent experiments. (**B**) ^3^H-Thymidine incorporation in cells expressing IL-16 specific shRNA or control cells. Significance was calculated by paired sample *t* test. (**C**) Cell cycle distribution of RPMI-8226 cells after IL-16 knockdown were determined by flow cytometry after staining with BrdU and 7-AAD. Bars indicate changes in populations compared to cells expressing an unspecific shRNA or cells in which IL-16-specific shRNA was not induced. Bars represent the mean and error bars the standard deviation from 3 independent experiments. (**D**) Clonogenic growth of a MM cell line RPMI-8226 14 days after induction of an IL-16-specific shRNA or a control shRNA as determined by light microscopy. Bars represent the mean and error bars the standard deviation of 6 independent experiments.

Since all of our analyses had pointed to a central role of IL-16 in supporting the proliferation of myeloma cells, we next analyzed the growth behavior of MM cells after stable IL-16 knockdown. Indeed, suppression of IL-16 expression led to a distinct reduction in cell culture growth (Figure 4A). Importantly, the reduced growth of MM cells after IL-16 knockdown was not due to enhanced apoptosis since IL16 silencing did not have an effect on cell viability (data not shown). Investigating the proliferative activity of myeloma cells after IL-16 silencing, however, we observed a significantly reduced cellular division rate (Figure 4B). Analyzing the cell cycle distribution of MM cells after IL-16 knockdown we observed a significant reduction of cells in G2 phase and a slight reduction of cells in S phase, while numbers of cells in G0/G1 phase had increased (Figure 4C).

Finally, we evaluated the effect of stable IL-16 silencing on the outgrowth of MM progenitor cells, which are thought to be responsible for the occurrence of chemotherapy-resistance and the high relapse rate in MM. As shown in Figure 4D, IL-16 knockdown dramatically impaired the clonogenic outgrowth of MM cells and cells lacking IL-16 expression completely lost their potential for self-renewal. These data suggest that IL-16 not only promotes the proliferation of conventional MM cells but that it is also indispensable for the growth of the MM progenitor population.

## DISCUSSION

Within the last decade the outcome of MM patients has dramatically improved but the disease remains incurable and the majority of the patients will succumb to the disease within 6 years after initial diagnosis emphasizing our incomplete understanding of the biology of MM. We suggest that more detailed knowledge on the factors promoting proliferation, survival, and chemotherapy resistance in MM will help us identify targets for novel therapeutic approaches which will lead to improved outcomes, and maybe even cures, in this fatal malignancy.

We have previously identified IL-16 as an important factor promoting the malignant phenotype of tumor cells from MM patients [15]. However, more detailed knowledge on the exact pattern of IL-16 expression, secretion, the molecular mechanisms behind its function in MM, and its effect on individual MM cell subtypes has been missing. Therefore, we decided to first investigate the pattern of IL-16 production and secretion by MM cells and we observed a periodic pattern of IL-16 synthesis. It remains to be determined if the approximately circadian rhythm of IL-16 expression that we have described for the first time in neoplastic plasma cells is comparable to the one observed in colorectal cancer where so-called “clock genes” create a complicated molecular time-keeping system consisting of multiple positive and negative feedback loops at transcriptional and translational levels. In the latter tumor type a circadian system coordinates and regulates multiple cellular processes implicated in cancer development such as metabolism, cell cycle, and DNA damage response [31].

Importantly, we found that the upregulated and periodic expression of IL-16 led to consistently high levels of the cytokine in the extracellular space. Interestingly, the extent of release of IL-16 from a given myeloma cell line correlated with its proliferative activity, suggesting that this cytokine might indeed be an important pro-proliferative factor in MM. Using transient IL-16 knockdown we had previously described a role of IL-16 in promoting MM cell proliferation [15], however, we wondered whether the oscillating pattern of IL-16 mRNA expression might have affected our initially reported effect sizes observed in functional assays. For our current analyses we, therefore, established an inducible knockdown system using lentiviral transduction of IL-16-specific shRNA constructs, enabling us to achieve a very efficient and, most importantly, stable mRNA and protein knockdown. Repeating our earlier experiments now using our stable knockdown system, we found most effect sizes of our initial analyses to be comparable to this new system. In particular we observed similarly reduced proliferation rates in IL-16 knockdown cells without any effect on cellular apoptosis. In addition, we now describe a significant reduction of cells in S and G2 phase, which is in contrast to earlier studies describing an inhibition of cell cycle progression by overexpressed Interleukin-16 in healthy immune cell subsets [32, 33]. This discrepancy may be caused by different factors, e.g. differential posttranslational processing, dysregulated autocrine signaling, or preferential use of alternative adapter molecules. Importantly, due to the difficulty of studying proliferation in primary human MM cells [34] our study focused on cell lines and does not take into account potential confounding factors present in primary MM samples.

To further corroborate our findings we analyzed whole genome expression in two MM cell lines using an mRNA-based microarray. Performing cluster analysis and gene ontology mapping we found that, as expected, the biological system most strongly influenced by IL-16 knockdown was cell proliferation followed by a comparably minor effect on the expression of genes related to cell death. We also observed differences in the number of regulated genes between the tested cell lines suggesting that despite comparable functional consequences IL-16 signaling in MM cells may occur through multiple molecular pathways. Validating our findings using RT-PCR based expression arrays we confirmed that multiple genes essential for three major molecular pathways in MM cell biology, PI3K, NFκB, and MAPK, were affected by IL-16 knockdown. Interestingly, in contrast to our previous report in which we described a strong upregulation of JUN and FOS after IL-16 knockdown, we now observed a moderate downregulation of these genes. JUN and FOS belong to the group of immediate-early genes that respond rapidly to various stimuli [35, 36] and the expression of which peaks only hours after the initiating event [37]. In the current experiment we performed expression analyses 5 days after continuous shRNA expression compared to 3 days after a single siRNA transfection in our previous article. Based on these initial data we hypothesize that even though JUN and FOS are highly expressed immediately after IL-16 knockdown, IL-16 loss does not lead to their sustained upregulation. We are currently exploring in more detail the downstream elements of IL-16 signaling in order to elucidate the mechanism by which secreted IL-16 affects MAPK and PI3K pathways MM, which have previously been shown to play an important role in in the etiology and maintenance of MM [38–42], rendering them promising therapeutic targets [43]. Out of all our analyses on the effects of stable IL-16 knockdown on MM cells, the most important observations were the consequences IL-16 withdrawal had on the clonogenic subpopulation within the bulk of tumor cells. Using the stable knockdown system we found that long-term IL-16 knockdown completely prevented clonogenic growth *in vitro*. This finding indicates an extraordinary dependence of clonogenic MM cells on the IL-16 pathway and is in line with a previous report linking IL-16 to the development of B cells in aging mice [44]. While this effect has not yet been demonstrated in humans and it remains unclear whether IL-16 contributes to the early development of MM the data point to an important role of IL-16 in maintaining healthy as well as malignant B cell populations.

In conclusion, establishing a stable and inducible knockdown system, and thereby minimizing the effect of oscillating mRNA expression on knockdown efficiency and functional assays, we confirmed the important role of IL-16 as a proliferation-inducing cytokine in myeloma. We further substantiated this finding by performing whole genome expression analyses. Most importantly, we were able to demonstrate that myeloma-propagating cells become entirely unable to form colonies in the absence of IL-16. Overall, our results demonstrate that tumor-regenerating MM cells may be particularly susceptible to IL-16 neutralization, suggesting an important role of anti-IL-16 therapies in the treatment of MM, in particular in a sequential combination with existing strategies targeting the bulk of MM cells.

## MATERIALS AND METHODS

### Cell lines

Cell lines AMO-1, EJM, IM-9, KMS-12-BM, LP-1, MOLP-8, NCI-H929, RPMI-8226, and U-266 were obtained from the German Collection of Microorganisms and Cell Cultures (DSMZ, Braunschweig, Germany). Cell line SKO-007 was provided by the New York branch of the Ludwig Institute for Cancer Research. All lines were maintained in RPMI-1640 medium containing 10% fetal calf serum, 50 U/ml Penicillin, and 50 μg/ml Streptomycin (Life Technologies, Carlsbad, CA) at 37°C in 5% CO_2_ humidified atmosphere. Virus-producing cell line HEK293T was a kind gift from Prof. Fehse (University Medical Center Hamburg-Eppendorf) and was maintained in complete DMEM medium (Life Technologies).

### Quantitative real-time PCR and PCR arrays

Total RNA extraction and purification was performed with the RNAeasy Mini Kit (Qiagen, Hilden, Germany). Quality and integrity of RNA was tested by photometric measurements and the Agilent RNA 6000 Nano Kit (Agilent Technologies, Santa Clara, CA), respectively. RNA for quantitative real-time PCR (qRTPCR) was transcribed using AMV reverse transcriptase (Promega, Madison, WI). Target gene expression was determined using SYBR Green Mastermix (Roche, Basel, Switzerland) over 40 PCR cycles. For IL-16 quantification, a commercially available primer mix (Qiagen) was used. Primers for GAPDH quantification were obtained from MWG Biotech (Eberberg, Germany): Forward: CCGAGCCACATCGCTCAGACAC; Reverse: AGCCTTGACGGTGCCATGGAAT. RNA for signal pathway analyses was transcribed using RT^2^ First Strand Kits. Pathway-focused gene expression was analyzed using RT^2^ Profiler^TM^ MAP kinase, NFκB and PI3 kinase real-time PCR arrays (Qiagen) according to the manufacturer's instructions and results were analyzed using the web-based RT^2^ Profiler^TM^ PCR Array Data Analysis Software.

### Gene expression profiling

Total RNA with a RNA integrity number (RIN) of at least 9.7 was used for genome wide-expression analyses performed by Atlas Biolabs (Berlin, Germany). Briefly, RNA was transcribed into cRNA and labeled with fluorophores via Ambion WT Expression Kit (Affymetrix, Santa Clara, CA). Labeled cRNA was hybridized with transcript specific oligonucleotides immobilized on a GeneChip Human Gene 2.0 ST Array (Affymetrix). Fluorescence signals of bound cRNA were detected by Affymetrix GeneChip Command Console Software. Raw data were transformed using Affymetrix Expression Console Software, including background correction, distribution-based quantile normalization, and logarithmic data transformation, via robust multi-array average algorithm (RMA method). The statistical analysis program R allowed for identification of differential gene regulation and cluster analyses. For assignment of regulated genes to biofunctional groups Ingenuity software was used.

### Enzyme-linked immunosorbent assay (ELISA)

Cells were plated at a density of 3 × 10^5^ cells/ml and supernatants were collected after 72 h and frozen at −80°C until final analysis. The IL-16 Quantikine ELISA (R&D Systems, Minneapolis, MN) was used to determine cytokine concentrations in supernatants.

### Enzyme-linked immunospot (ELISPOT) assay

To quantify the number of IL-16-secreting cells the Human IL-16 ELISpot Development Module (R&D Systems) was used. A hydrophobic 96well PVDF membrane plate was covered with an IL-16 specific capture antibody. Cells were plated in a decadal dilution series on captured PVDF membranes while being in their optimal growth phase. Following a 16 h incubation period, cells were removed from the PVDF membrane and IL-16 was detected using an IL-16-specific biotinylated antibody. Enzymatic conversion of BCIP/NBT created visible spots indicating single IL-16-secreting cells.

### Western blot

Cells were treated with cell lysis buffer (Biovision, Milpitas, CA) including a protease inhibitor cocktail (Roche) for 20 min on ice. Lysed cells were centrifuged for 20 min at 12,000 rcf and supernatant was collected and frozen at −80°C until final analysis. An appropriate amount of protein was applied per lane for immunoblot analysis. Free binding sites of the nitrocellulose membrane containing the cellular proteins were blocked with blocking buffer (5% milk powder in TBS-T). Primary murine antibodies were used at a 1:1,000 (anti-human IL-16, clone 70719; R&D Systems), 1:3,000 (anti-human β-catenin, clone H102; Santa Cruz), and 1:5,000 dilution (anti-human GAPDH, clone 6C5; Santa Cruz), respectively. The secondary HRP-labeled antibody (R&D Systems) was used at a 1:5,000 dilution and signals were detected using chemiluminescent ECL solution.

### Flow cytometry

For intracellular staining cells were washed once with DPBS (Life Technologies), fixed for 20 min with IC fixation buffer (eBioscience, San Diego, CA), and then washed twice with permeabilization buffer (Biolegend, San Diego, CA). Intracellular staining was performed with PE-labeled IL-16-specific antibody clone 14.1 (BD Biosciences, Heidelberg, Germany) and FITC-labeled GAPDH-specific antibody clone FL-335 (Santa Cruz). For assessment of apoptosis cells were washed once with DPBS and then treated with Life/Dead Fixable Dead Cell Stains (Life Technologies) at a 1:100 dilution for 30 minutes. Cells were stained with APC-labeled Annexin V (Cell Signaling Technology, Danvers, MA) in Annexin Binding Buffer (BD Biosciences) for 15 min. For cell cycle analyses cells were analyzed using a BrdU Kit (BD Biosciences) after synchronization by withdrawal of FCS for 16 h. Within the last 4 h of cell culture thymidine analog BrdU was added. Cells were washed, fixed, and permeabilized. Following DNAse digestion, the DNA of cells was stained with BrdU-specific APC-labeled antibody for 20 min and with 7AAD for 15 min, respectively. Cells were then analyzed by flow cytometry.

### Lentivirus production, IL-16 gene silencing, and analysis of IL-16 promoter activity

Lentiviral particle-containing supernatants were generated by transient calcium-phosphate transfection of HEK293T packaging cells using an expression vector, a third generation packaging plasmid mix, and VSVG pseudotyping. In brief, 15 μg of expression plasmid, 10 μg pMDLg/pRRE, 5 μg pRSVRev, and 2 μg pVSVG were mixed in 500 μl of a 0.25 M CaCl_2_ solution. Dropwise, equal volumes of 2x HEPES-buffered saline were added to the plasmid mix. After incubating for 20 min this mix was added to the packaging cells. 8 h after incubation, cell culture medium was exchanged for fresh medium. Cell-free viral supernatants were collected 24 h and 48 h after transient transfection. Silencing of IL-16 gene expression was performed by lentiviral transduction of shRNAmir using the TRIPZ vector system (Open Biosystems, Huntsville, AL). MM cell lines were infected at an MOI of 5 or 10, respectively. The following IL-16-specific shRNAmir sequences were used: shRNAmir1: sense: CCACGATTGTCATCAGGAGAAA, antisense: TTTCTCCTGATGACAATCGTGA; shRNAmir2: sense: CGGCATCCATGTCACCATCTTA, antisense: TAAGAT GGTGACATGGATGCCG; shRNAmir3: sense: ACAGCA AAAGGTTGTTCCTAAA, antisense: TTTAGGAACA ACCTTTTGCTGGT, shRNA4: sense: AGCTCCTAACC TTCCTGTAAAC, antisense: GTTTACAGGAAGGTT AGGAGCC, unspecific shRNA: no sequence specifications. The expression of shRNAmir was induced by adding doxycycline to the cell culture. For functional validation of IL-16, two controls were applied. Uninduced controls transduced with IL16 specific shRNAmir without adding doxycycline and unspecific control cells transduced with unspecific shRNAmir and treated with doxycycline.

In addition, three lentiviral vectors encoding destabilized GFP constructs [28] based on pTurboGFP-dest1 (Evrogen, Moscow, Russia) for the analysis of IL-16 promoter activity were generated. Mutations H423A, and D433A/D434A were introduced into TurboGFP using the QuickChange II Site-Directed Mutagenesis kit (Agilent). The IL-16 promoter was amplified from primary human genomic DNA using these primers: F: GCAGCTCAAT ATCCGTTTTTCCGTC; R: GCTGGCTCTTCTCCACC CTGG (Eurofins MWG Operon, Ebersberg, Germany). Both the promoter and GFP were cloned into the ViraPower HiPerform Promoterless Gateway vector (Invitrogen, Carlsbad, CA, USA) using pENTR vectors. Lentiviral supernatants were prepared according to the manufacturer's instructions and MM cell lines were transduced as described above. MM cell lines were synchronized by serum starvation for 2 h and fluorescence intensity was monitored in 15 min intervals for 65 h using a LiveCell Spinning Disk system (Improvision, Coventry, UK).

### 3H-Thymidine incorporation assay

Cells were washed twice with PBS, seeded in duplicates at 2×10^5^ cells/ml in a 96 well plate and treated with increasing concentrations of an IL-16 neutralizing antibody (clone 14.1, EMD Millipore, Darmstadt, Germany) or an isotype control antibody (Millipore). After 12 h cells were harvested and analyzed on a beta scintillation counter.

### Statistical analyses

Statistical analyses were performed using GraphPad Prism 6.0 (GraphPad Software Inc., La Jolla, CA). Paired *t*-tests were applied to determine levels of significance.
